# Targeting Adiposity and Inflammation With Movement to Improve Prognosis in Breast Cancer Survivors (The AIM Trial): Rationale, Design, and Methods

**DOI:** 10.3389/fonc.2022.896995

**Published:** 2022-06-20

**Authors:** Dong-Woo Kang, Rebekah L. Wilson, Paola Gonzalo-Encabo, Mary K. Norris, Marybeth Hans, Meghan Tahbaz, Jackie Dawson, Danny Nguyen, Amber J. Normann, Alexandra G. Yunker, Nathalie Sami, Hajime Uno, Jennifer A. Ligibel, Steven D. Mittelman, Christina M. Dieli-Conwright

**Affiliations:** ^1^ Department of Medical Oncology, Dana-Farber Cancer Institute, Boston, MA, United States; ^2^ Department of Medicine, Harvard Medical School, Boston, MA, United States; ^3^ Division of Breast Surgery, Brigham and Women’s Hospital, Boston, MA, United States; ^4^ Department of Medicine, Morsani College of Medicine, University of South Florida, Tampa, FL, United States; ^5^ Department of Physical Therapy, California State University, Long Beach, Long Beach, CA, United States; ^6^ Department of Health Sciences, Boston University, Boston, MA, United States; ^7^ Department of Nutrition, Harvard T.H. Chan School of Public Health, Boston, MA, United States; ^8^ Department of Medicine, University of Southern California, Los Angeles, CA, United States; ^9^ Children’s Discovery and Innovations Institute, University of California, Los Angeles, Los Angeles, CA, United States

**Keywords:** exercise, circuit training, randomized controlled trial, obesity, chronic inflammation, adipose tissue, breast cancer

## Abstract

**Background:**

Obesity is a significant contributor to breast cancer recurrence and mortality. A central mechanism by which obesity stimulates cancer progression is through chronic, low-grade inflammation in adipose tissue. Exercise interventions to target chronic inflammation has a potential to improve obesity- and breast cancer-related outcomes; however, no studies have investigated the roles of exercise in modulating adipose tissue inflammation in breast cancer survivors. Also, it is unclear which exercise prescription would be optimal to maximize the outcomes. Therefore, we designed a randomized controlled trial (Taking AIM at Breast Cancer: Targeting Adiposity and Inflammation with Movement to Improve Prognosis in Breast Cancer Survivors [AIM] Trial) to examine the mechanisms by which different modalities of exercise impact chronic inflammation as a biomarker of breast cancer prognosis.

**Methods:**

The AIM trial is a prospective, three-armed, phase II randomized controlled trial investigating the effects of a 16-week supervised circuit aerobic and resistance exercise (CARE) program versus a traditional aerobic and resistance exercise (TARE) program and attention control (AC) on adipose tissue inflammation in breast cancer survivors. 276 patients who are diagnosed with stage 0-III breast cancer, post-treatment, sedentary, and centrally obese are randomized to one of the three groups. The CARE and TARE groups participate in thrice-weekly supervised exercise sessions for 16 weeks. The AC group are offered the CARE program after the intervention period. The primary endpoint is adipose tissue inflammation assessed by core biopsy and blood draw. The secondary and tertiary endpoints are sarcopenic obesity, physical fitness and function, and patient reported outcomes. The exploratory outcomes are long-term breast cancer outcomes.

**Discussion:**

This is the first randomized controlled trial examining the effects of exercise on adipose tissue inflammation in obese, breast cancer survivors. Our findings are anticipated to contribute to a better understanding of exercise modalities and mechanisms on adipose tissue inflammation that can potentially improve breast cancer prognosis.

**Clinical Trial Registration:**

https://clinicaltrials.gov/ct2/show/NCT03091842 identifier [NCT#03091842].

## 1 Introduction

Obesity and breast cancer represent two common diseases globally with increasing prevalence worldwide (1). In the United States, 2 in 3 adults are overweight or obese and 1 in 8 women will develop breast cancer, where each independently has a profound impact on public health (2, 3). Notably, over 50% of breast cancer patients are considered overweight or have obesity (i.e., body mass index ≥ 25.0 kg/m^2^) at diagnosis, and 50-96% of them are experiencing further weight gain during cancer treatment (4–6). Moreover, obesity increases the risks of breast cancer recurrence and all-cause and cancer-specific mortality up to 40% (7, 8). A central mechanism by which obesity stimulates cancer progression is through chronic, low-grade inflammation in adipose tissue, particularly in white adipose tissue (WAT) (9–12). WAT is comprised of metabolically active adipocytes capable of secreting adipokines and proinflammatory cytokines related to tumorigeneses (9–12). Given obesity-induced inflammation in adipose tissue leading to physiological disruptions (13, 14), there is an urgent need for non-pharmacological strategies that target obesity-related inflammation to improve prognosis in breast cancer survivors.

Current recommendations by the American College of Sports Medicine (ACSM) (15, 16) encourage cancer survivors to perform a minimum of 150 minutes per week of moderate-intensity or 75 minutes per week of vigorous-intensity aerobic exercise (AE), as well as two resistance exercise (RE) sessions per week involving all major muscle groups. Considering these guidelines, AE combined with RE, referred here as “traditional aerobic and resistance exercise” (TARE), represents an effective approach to exercise prescription in breast cancer survivors, as combined programs improve systemic inflammation, metabolic diseases, and muscular strength (17–19). On the other hand, “circuit aerobic and resistance exercise” (CARE) is an innovative training program, incorporating interval and circuit training and periodization, and it has been shown to improve muscle and cardiorespiratory fitness among breast cancer survivors (20) and bone health among female cancer survivors (21). However, it is unclear which exercise program would be optimal to improve adiposity and inflammatory outcomes in breast cancer survivors.

**Figure 1:**
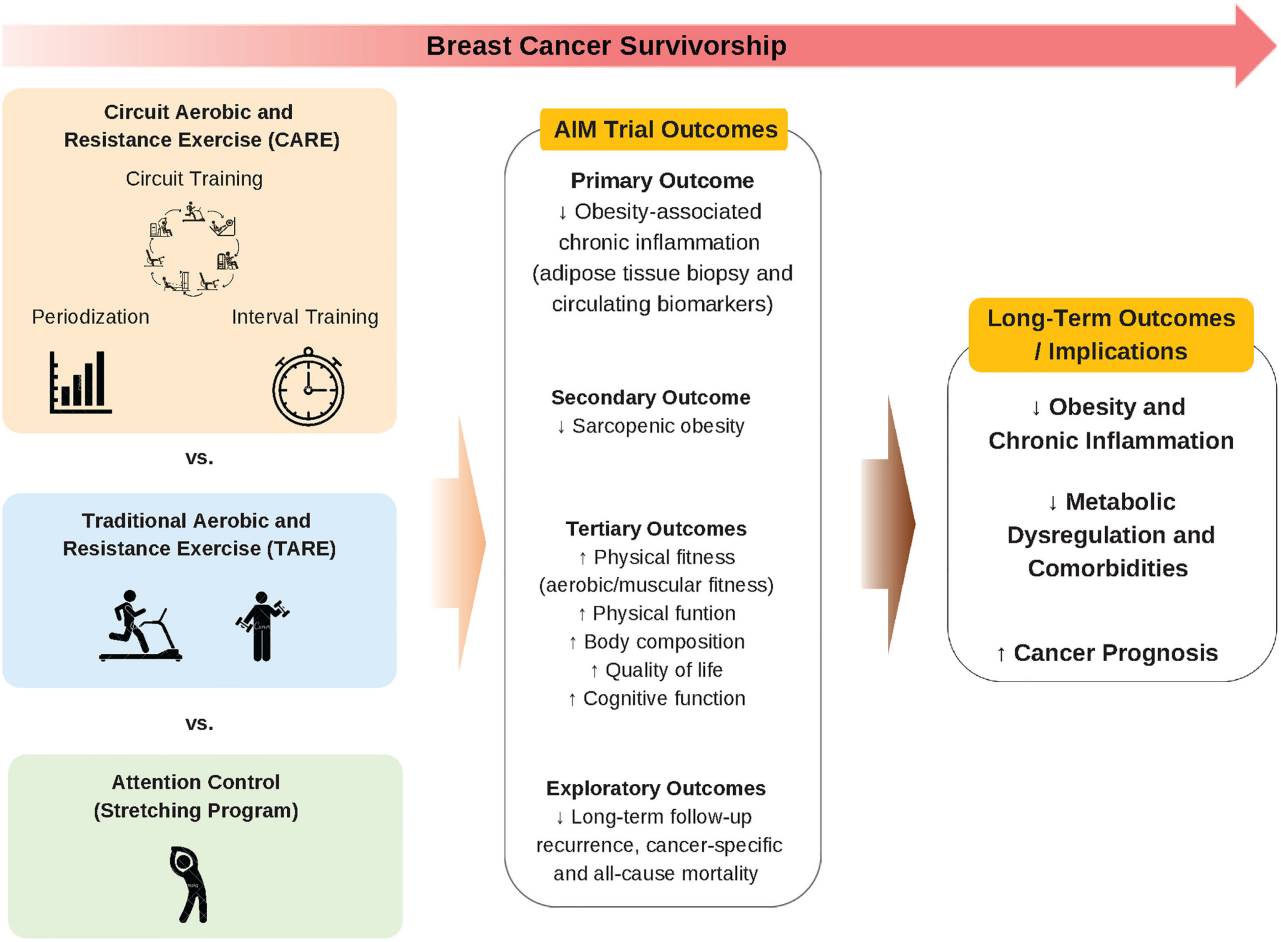
Conceptual framework of the AIM trial.

Hence, we designed a randomized controlled trial, “Taking AIM at Breast Cancer: Targeting Adiposity and Inflammation with Movement to Improve Prognosis in Breast Cancer Survivors: the AIM trial”, as the first intervention study to compare the impact of different modalities of exercise on chronic inflammation as a biomarker of breast cancer prognosis (**Figure 1**). The primary objective of the AIM trial is to investigate the effects of CARE vs. TARE training programs on adipose tissue-driven chronic inflammation in breast cancer survivors. The secondary and tertiary objectives are to examine the intervention effects on sarcopenic obesity, physical fitness and function, cognition, and quality of life. Finally, the exploratory objective is to examine the long-term clinical outcomes. We hypothesize that CARE will reduce chronic inflammation as well as improve secondary and tertiary outcomes to a greater degree compared to TARE and attention control (AC).

## 2 Methods And Analysis

### 2.1 Study Design

**Figure 2:**
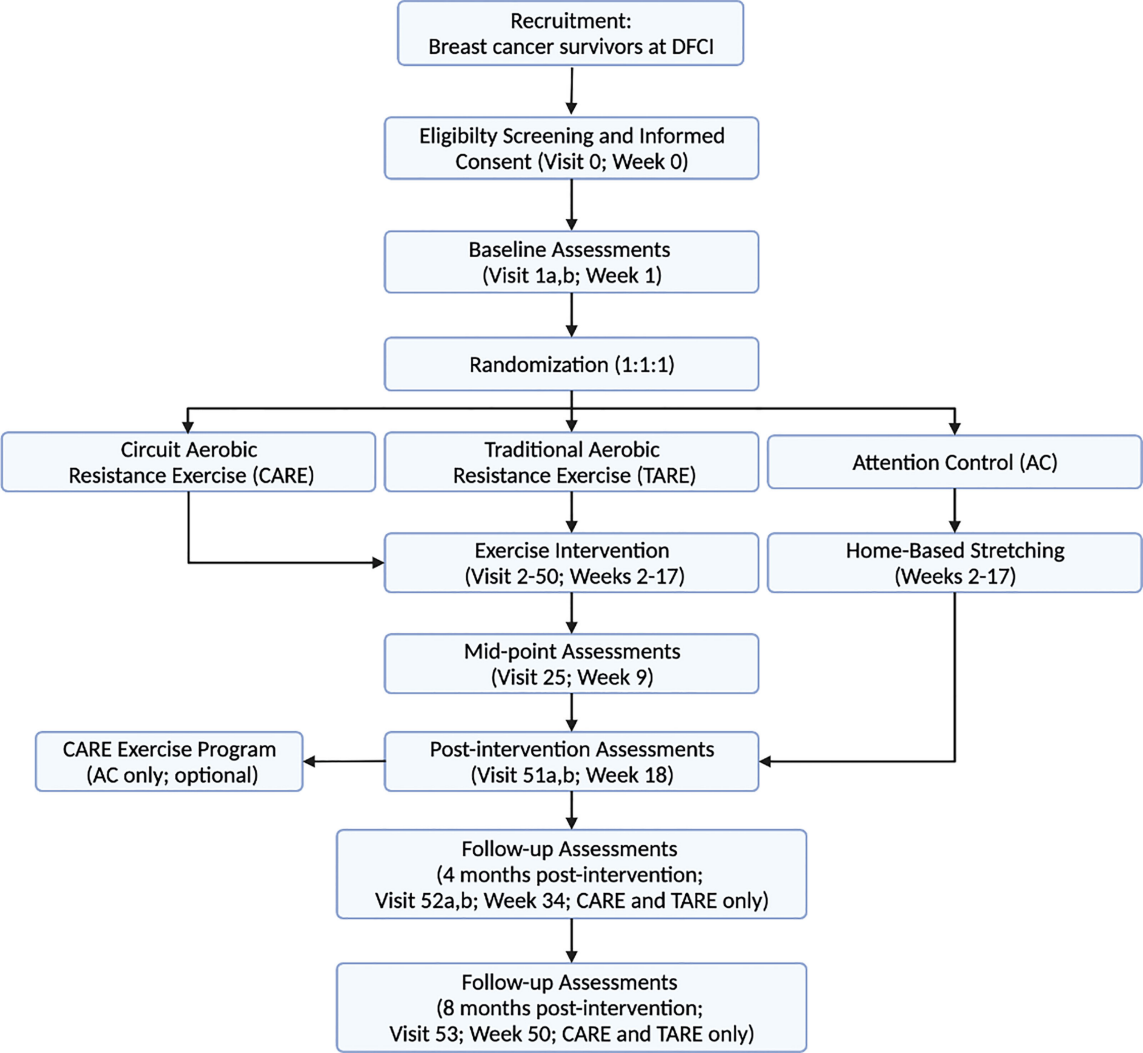
CONSORT flow diagram of the AIM trial.

**Figure 3:**
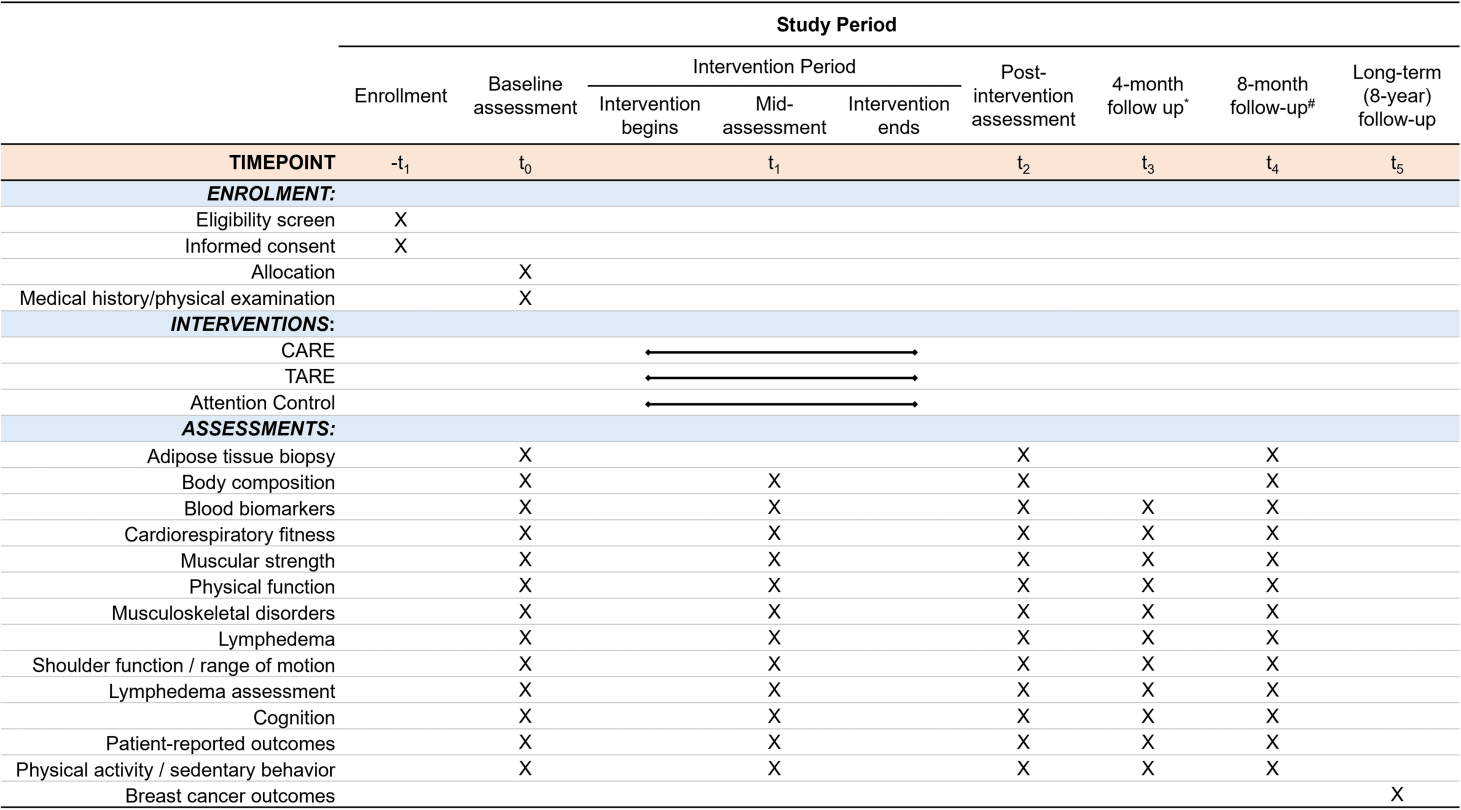
Study timeline of the AIM Trial. CARE, Circuit Aerobic and Resistance Exercise; TARE, Traditional Aerobic and Resistance Exercise; AC, Attention Control. All three groups are assessed otherwise noted. Follow-up timepoints are defined as the period from the post-intervention assessments. ^*^Participants in the AC group who do not choose to perform the supplementary CARE program after the intervention period will not complete selected assessments testing at this time point. ^#^Participants in the AC group will not complete assessments at this time point.

The AIM study is a prospective, single-center, three-arm, phase II randomized controlled trial conducted at the Dana-Farber Cancer Institute, Boston, MA. A total of 276 breast cancer survivors with obesity and have completed chemotherapy and/or radiation are randomly assigned to one of three groups: CARE, TARE, or AC. The study involves a 16-week intervention with 4- and 8-month follow-up periods. This study protocol is in accordance with the Consolidated Standards of Reporting Trials guidelines (22), and the study schema and timeline are illustrated in **Figures 2** and **3**, respectively. This study is approved by the Institutional Review Board (IRB #20-172) and registered at ClinicalTrials.gov (NCT#03091842).

### 2.2 Participants and Recruitment

This trial includes women with newly diagnosed stage 0-III breast cancer who have completed cancer-related treatment, and the detailed inclusion and exclusion criteria are provided in **Table 1**. Various recruitment strategies are used to identify potential participants including screening breast clinic lists, use of patient advertisements in waiting areas and through online platforms and newsletters. Once potential participants are identified through the patient list, a study coordinator contacts the patients’ treating providers or oncologists and request permission to contact the patients to invite them to participate in the study. Potentially eligible participants are further screened by study staff either in person or by phone to confirm other eligibilities, including current exercise levels using the Godin Leisure Time Questionnaire (23) and past and current medical conditions using the Physical Activity Readiness Questionnaire (PAR-Q). Interested participants are then scheduled for a visit with a member of the study staff to review the protocol and sign informed consent. Due to the ongoing COVID-19 pandemic, we have also adopted the use of electronic consent (e-consent) to obtain consent remotely.

**Table 1:** Inclusion and exclusion criteria of the AIM Trial.

Inclusion Criteria	Exclusion Criteria
• Over 18 years of age • Newly diagnosed women with stage 0-III breast cancer, low grade disease positive for estrogen and progesterone receptors • Having undergone lumpectomy or mastectomy, (neo)adjuvant chemotherapy and/or radiation therapy • Not receiving concurrent cancer treatment • Centrally obese with the following criteria: BMI >30 kg/m^2^ or body fat >30% (as measured by bioimpedance analysis), waist circumference >35 inches • Currently participating in less than 60 minutes of structured exercise per week • Without any cardiovascular, respiratory, or musculoskeletal disease or joint problems that preclude exercise • Not currently or planning to become pregnant • Speaking English	• Metastatic disease or other active malignancies • Currently receiving any other investigational agents, or concurrent biological, chemotherapy, or radiation therapy • Not completed any of breast cancer treatment (e.g., surgery, chemotherapy, or radiation) • Having uncontrolled illness including ongoing or active infection, uncontrolled diabetes, hypertension, or thyroid disease that preclude participation in exercise • Participates in more than 60 minutes of structured exercise/week • Is planning reconstructive surgery with flap repair during trial and follow-up period • Smoker • Unable to travel to research facilities

### 2.3 Randomization and Blinding

Upon the completion of baseline assessments, patients are randomly assigned to either CARE, TARE, or AC in an equal allocation ratio (1:1:1) using a permuted-block design with varying block sizes. The randomization is stratified by menopausal status (premenopausal and postmenopausal) which is evaluated at time of diagnosis. A study biostatistician (H.U.) prepares the randomization schema prior to trial start-up, and the randomization allocation is provided to staff *via* a web-based application (REDCap) (24), and study investigators are blinded to the randomization process. The intervention allocation is not blinded to the study participants, interventionists, and outcome assessors due to the nature of exercise intervention.

### 2.4 Exercise Intervention

**Figure 4:**
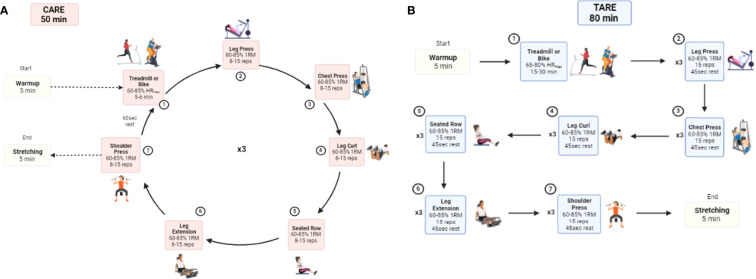
Comparison of CARE **(A)** and TARE **(A)** protocols for an exercise session. CARE, Circuit Aerobic and Resistance Exercise; TARE, Traditional Aerobic and Resistance Exercise; HRmax, maximum heart rate; 1RM, 1 repetition-maximum.

The CARE and TARE groups receive a 4-month exercise intervention at an exercise facility located within the cancer center, and all exercise sessions are supervised one-on-one by a clinical exercise trainer. Both exercise programs consist of a total of 48 sessions, provided three times per week for 16 weeks for approximately 60 minutes per session. Heart rate (HR) is monitored throughout all training sessions using a Polar^®^ heart monitor (Lake Success, NY). Each session begins with a 5-minute warm-up of low-intensity AE and concludes with 5 minutes of the same 3-4 static stretches conducted by the AC group, which will target major muscle groups, each held for 30 seconds. One-repetition maximum (RM) and peak oxygen consumption (VO_2peak_) obtained during baseline testing are used to determine resistance load for each exercise and aerobic intensity, respectively. The details of a single exercise session of each CARE and TARE program are illustrated in **Figure 4**.

#### 2.4.1 Circuit Aerobic and Resistance Exercise (CARE)

The CARE intervention is a systematically progressed AE and RE program. The intensity of exercise begins at a low intensity and gradually increases to high intensity over the 4-month mesocycles to optimally elicit improvements in body composition, physical function, and cardiometabolic makers (25). The details of the linear periodization for the CARE program are described in **Table 2**. In brief, the first mesocycle, the preparatory period, is performed at moderate intensity (60-65%) to increase tolerance of higher training intensities that occur later in the program (26). The majority of the program (weeks 5-12, mesocycles 2-3) are performed at moderate-to-vigorous intensity (67-80%) to emphasize increases in lean mass (27) and decreases in fat mass (28), with a hypertrophy training goal during RE (26), and intensities approaching 80% VO_2peak_ during AE (28). The fourth mesocycle incorporates higher intensities (80-85%), which are necessary to further obtain improvements as performance increases (29). The volumes of the RE decrease throughout the intervention period to accommodate the increase in intensity, while the volumes of the AE increase to match the volumes performed in the other exercise group. AE is performed on a treadmill or a bike, and RE consists of 6 exercises, including leg press, chest press, leg curl, seated row, leg extension, and shoulder press (lateral raise or scaption will be substituted if participants are unable to press overhead), using exercise machines and dumbbells. Each upper- and lower-body exercise is performed in an alternating manner to provide active recovery periods for the exercised muscle groups between each exercise and to constitute circuit-style training. Specifically, participants begin with AE, then alternate 3 lower-extremity and 3 upper-extremity REs that target major muscle groups with no rest between RE exercises. After completion of a set of exercises in one circuit, participants return to AE to start the next set after 1-minute rest between sets, until all prescribed sets are completed.

**Table 2:** Periodization model for exercises in the CARE program.

Training Period	Resistance Exercise		Aerobic Exercise	
	Intensity (%1RM)	Volume (repetitions x sets)	Intensity (%HR_max_)	Volume (duration x sets)
**Mesocycle 1** **(Preparatory)**				
Weeks 1-2	60%	15 x 3	60%	5 min x 3
Weeks 3-4	65%	12-15 x 3	65%	5 min x 3
**Mesocycle 2** **(Moderate Intensity)**				
Weeks 5-6	70%	10-12 x 3	70%	5 min x 4
Weeks 7-8	70-67%	10-12 x 3	75%	5 min x 4
**Mesocycle 3** **(Vigorous Intensity)**				
Weeks 9-10	70%	10 x 3	80%	5 min x 5
Weeks 11-12	75%	8-10 x 3	80%	5 min x 5
**Mesocycle 4** **(High Intensity)**				
Weeks 13-14	80%	8 x 3	85%	6 min x 5
Weeks 15-16	85%	8 x 3	85%	6 min x 5

RM, repetition maximum; x, times (e.g., 8 x 3 = 8 repetitions completed three times); HR_max_, maximum heart rate; min, minutes.

#### 2.4.2 Traditional Aerobic and Resistance Exercise (TARE)

Patients assigned to the TARE group begin training sessions with AE on the treadmill or bike, followed by a total body RE with the same 6 exercises as in the CARE intervention. The duration and intensity of AE are progressing from 15 to 30 minutes and from 65 to 80% of HR_max_, respectively. The initial RE will be 60% 1RM and will increase by 10% when a participant is able to complete three sets of 10 repetitions in two consecutive sessions. Each session consists of AE and 3 sets of each RE with a 45-second rest period are taken between each RE set, which takes approximately a total of 80 minutes. Both the AE and RE are matched in volume to that of the CARE program.

#### 2.4.3 Attention-Control (AC)

Patients randomized to the AC group complete a 16-week home-based stretching program with the same stretches in the CARE and TARE groups at the end of their sessions. Minimal caloric expenditure is expected to be incurred as the stretches are low-intensity, low-impact, and low-volume. Participants are given an instructional booklet and trained in how to use the booklet to maximize the standardization of the home-based stretching (30) and increase compliance (31). Participants are also asked to complete weekly records detailing flexibility compliance and any physical activity performed outside of the study. At the end of the initial 16-week period, the AC group will be offered the 16-week CARE program weeks. Participants in the AC group who choose to perform the CARE program will complete one more assessment after the exercise program. Those who do not choose to partake in the CARE program will be finished with the study after the initial 16-week period.

### 2.5 Outcome Measures

The outcomes are assessed at baseline, mid-intervention, and post-intervention with 4- and 8-month follow-up periods. All measures are performed by trained study personnel at the Dana-Farber Cancer Institute.

#### 2.5.1 Primary Outcomes

##### 2.5.1.1 Adipose Tissue Chronic Inflammation

M1 and M2 phenotypes are the primary outcomes. As the M1/M2 system alone does not accurately represent the heterogeneity of adipose tissue macrophages (ATMs) in obesity (32), we also examine secretion levels of inflammatory cytokines to provide a comprehensive examination of the presence of chronic adipose tissue inflammation, which include interleukin (IL)-6, IL-8, tumor necrosis factor-α (TNF-α), high-sensitive c-reactive protein (hs-CRP), leptin, and adiponectin. Participants can volunteer to undergo an abdominal subcutaneous fat biopsy under local anesthesia performed by a certified physician assistant (M.H.) and are asked to refrain from foods for 12 hours and anti-inflammatory pharmaceuticals for 3-5 days prior to biopsy. Participants lie supine on the examining table while the biopsy site is prepped and draped in a sterile fashion. Under ultrasound guidance performed by a licensed technician, a local anesthetic is injected into subcutaneous abdominal fat (midway between the iliac crest and umbilicus) after which a small skin nick is made. The core biopsy device equipped with a 13-gauge needle is inserted at the incision site.

Approximately 4 samples are taken from a single site, roughly a total of 40mg to 120mg of fat tissue. The biopsy is taken at the Brigham and Women’s Hospital and transported to the Immune Assessment Laboratory at the Center for Immuno-Oncology for processing. One sample is flash-frozen in liquid nitrogen and stored until RNA isolation, and the rest of the tissue is digested with a collagenase solution to create a single-cell suspension. Appropriate batch testing is conducted by research staff blinded to randomization.

##### 2.5.1.2 Systemic Inflammation

Fasting blood is taken from the antecubital vein by a trained phlebotomist following the biopsy procedure. For each sample, two 10 ml tubes are collected into EDTA and Serum tubes, along with one additional lavender topped tube (whole blood in EDTA). Samples are centrifuged and then aliquoted and stored at -80°C for future measures. Blood samples are then transported to the Brigham Research Assay Core for batch analyses for IL-6, IL-8, TNF-a, and hs-CRP. Other related systemic biomarkers such as leptin, adiponectin, insulin resistance, insulin-like growth factor (IGF)-1, IGF binding protein (IGFBP)-3, lipid profile, estradiol, sex hormone-binding globulin (SHBG), and free testosterone are also analyzed through commercially available blood testing kits.

#### 2.5.2 Secondary Outcomes

##### 2.5.2.1 Sarcopenic Obesity and Body Composition

Sarcopenic obesity is determined by body composition measured by dual-energy X-ray absorptiometry (DXA; *Hologic Inc., Marlborough, MA*). Appendicular fat-free mass (AFFM) is measured from a whole-body, hip, and spine scan, and predicted AFFM is calculated using the following equation: -14,529 + (17,989 * height in meter) + (0.1307 * total fat mass in kilograms) (33). A residual value of ≤-3.4 is defined as an individual having sarcopenic obesity (33). Lean mass, fat mass, and body fat % are also obtained from the whole-body DXA scan. A constant-tension tape measure is used to obtain waist circumference, defined as the distance around the waist using the umbilicus as the reference point, and hip circumference, defined as the distance around the widest girth of the buttocks using the greater trochanter as a landmark. Body composition is also assessed *via* bioelectrical impedance using a validated device (*Tanita 780, Arlington Heights, IL*) (34). The device estimates body fat using an algorithm based on their age, sex, height, and body weight.

#### 2.5.3 Tertiary Outcomes

##### 2.5.3.1 Cardiorespiratory Fitness

Cardiorespiratory fitness is assessed as VO_2peak_ during exercise exertion testing on a treadmill using the modified Bruce protocol (35). The test begins with a 5-minute warm-up, and a speed and incline increase every three minutes, concluding when the participant reached physical exhaustion and is unable to continue on the treadmill. A metabolic cart (*TrueOne 2400, ParvoMedic Inc., Salt Lake City, UT*) is used to measure gas exchange during the test, and VO_2peak_ is determined as the highest O_2_ value obtained at the maximal exertion. Heart rate (*Polar USA, Lake Success, NY*) and rating of perceived exertion (Borg CR10 scale) (36) are measured during the test. Results from this test are used to prescribe the target AE intensity. The 6-minute walk test is also conducted to assess aerobic endurance and capacity, which has been validated and widely used in cancer survivors (37, 38). Participants are instructed to walk as quickly as possible without running on an indoor pre-measured walkway for six minutes. Distance achieved during the 6-minute walk test is recorded in meters.

##### 2.5.3.2 Muscular Strength

Muscular strength is measured using one-RM. One-RM values are calculated and estimated from 10-RM using validated equations (39, 40). The test is conducted on a total of six exercises that are included in the intervention: 1) leg press, 2) leg extension, 3) leg curl, 4) chest press, 5) seated row, and 6) seated shoulder press (*Matrix Fitness, Cottage Grove, WI*). Following a warm-up, 3 attempts are given to reach the final 10-RM load with a 2-minute rest period between attempts. Grip strength is also assessed using a hand-held dynamometer on the participant’s dominant hand (*Camry Digital Hand Dynamometer, El Monte, CA*). In a standing position, participants grip the dynamometer handle with their arms down. Participants complete the test three times with the highest result recorded for analysis.

##### 2.5.3.3 Physical Function

Lower extremity physical function is assessed using the Short Physical Performance Battery (SPPB), which has been widely used and validated within the breast cancer population (41). This battery included three sections: 1) Timed balance is assessed with both feet side-by-side, semi tandem, and full tandem. These positions are held for 10 seconds with no external support or when the participant steps out of position. Participants have one attempt to complete the test, and the time in each position is recorded in seconds. 2) Gait speed is assessed over a 4-meter distance. Participant has two attempts and is asked to walk at their “usual” pace with the fastest time recorded in seconds. 3) Chair stand is assessed by recording the time in seconds that the participants need to complete five chair stands with only one attempt after a familiarization practice to ensure proper technique. Each section of the SBBP is given a score based on performance to then provide a total score.

Mobility and immediate fall risk are assessed using the Timed Up and Go (TUG) test (42). Participants start and end in a seated position assessing the time needed to walk around a cone placed 3 meters away, with three attempts to complete the test and one familiarization practice. The final time recorded in seconds is the average of the three attempts.

Gait speed is also assessed over a 6-meter flat surface. The time in seconds to walk the 6-meter surface at the participant’s “usual” and “fast” pace is recorded (43).

Functional power is measured using the Margaria Stair Climb test that has been previously used and associated with lower-extremity power and mobility in older adults (44). Participants walk/run up 10 stairs as fast, and safely, as possible. The time in seconds that the participant takes from the 3^rd^ stair to the 9^th^ stair is recorded, with three attempts given and a familiarization practice, where the average time is recorded.

Lastly, the sit-to-stand test is performed, with participants completing as many sit-to-stands as possible in 30 seconds. Participants are asked to rise to a full standing position. Only one attempt is given, and the number of total repetitions completed with the proper technique is recorded.

##### 2.5.3.4 Musculoskeletal Disorders

Peripheral neuropathy is assessed using two different tests (45–47). First, the Semmes-Weinstein Monofilament Examination (SWME), where participants are seated in an upright position with their socks and shoes removed and eyes closed. The SWME evaluates protective sensation at the pad of the great toe (foot), and at the pad of the index finger (hand). A nylon monofilament will be used and applied four times in each site. Second, vibration sensation is assessed by applying a tuning fork to the bony prominences of the great toe, thumb, and medial malleolus where the participant will verbally identify if the vibration stopped. The correct identification of the side of the body where the nylon monofilament was applied, and on/off vibration identification will be recorded.

##### 2.5.3.5 Lymphedema

Geometric arm volume calculations are performed to assess lymphedema on both upper limbs (48). We calculate arm volume using circumferential measurements taken at anatomic landmarks (48). The anatomic landmarks include the axillary fold, halfway between the axillary fold and antecubital fossa, antecubital fossa, halfway between antecubital fossa and wrist, and wrist. This method was determined to be a reliable and valid method of limb volume measurement. The participant will be supine, circumferential measurements will be taken with a constant tension tape measure. Calculations for limb geometric volume are completed using the frustum (truncated cone) volume (48). The interrater reliability for this method is high at >0.98 (49). We will calculate a percentage difference between the lymphedema limb and the uninvolved limb to examine the amount of lymphedema. Lymphedema is defined as a greater than 10% difference in volume calculation for the arm compared to the uninvolved upper extremity (48).

##### 2.5.3.6 Shoulder Range of Motion and Function

Shoulder active range of motion (AROM) is measured on both upper limbs. AROM is assessed in external rotation at 0°, external rotation at 90°, forward flexion, and abduction (50, 51), using a goniometer (*Jamar E-Z Read, Patterson Medical, Warrenville, IL*). Participants are standing in all tests except for external rotation at 90° where the participant is lying supine on the ground. Participants are asked to actively perform the movement tested. Participants perform the test three times after a familiarization trial where the tester passively moves the participant’s limb. The average of the three attempts is recorded in degrees.

The Y Balance Test Kit (*Move2Perform, Evansville, IN*) is used to assess upper body function (52). Starting position for the test is in a three-point plank on toes or knees depending on ability. The upper limb that is tested is placed on the main block and the non-tested limb is placed on the medial block. Feet are placed shoulder-width apart. Then, participants use their non-tested limb to move each block one at a time in the medial, inferolateral, and superolateral directions. The block must be moved in a controlled manner as far as possible, returning to the starting position once each block has been moved. Participants have three trials on each upper limb with 30 seconds rest between trials. The highest value obtained in each direction is used to calculate an average composite score.

Upper body strength and power are measured using the seated medicine ball throw (SMBT) (53). The starting position for this test is sitting on the floor with head, shoulders, and back against the wall and legs extended or bent depending on ability. The 2 kg medicine ball is held with both hands and covered in chalk. A measuring tape will be placed on the floor and stretched out over 10 meters. Participants will throw the medicine ball forward, in a straight line, as far as possible with head, shoulders, and back maintaining full contact with the wall. Participants have three trials and one familiarization trial with 60 seconds rest between each trial. To calculate a relative throwing distance, arm reach distance is measured from the wall in meters and then subsequently deducted from the total throwing distance. The average of the throwing distances is recorded in meters.

We assess upper body function using the closed kinetic chain upper extremity stability test (CKCUEST) (54). Starting position for the test is in a three-point plank on toes or knees depending on ability with hands 91.4 cm (36 in.) apart (marked with two stripes of tape on the floor) and with both shoulders perpendicular to their hands and feet hip-width apart. Participants use their dominant hand to reach across the body, touch the non-dominant hand, and return to the starting position. Then, they perform the movement with the non-dominant hand. Participants are asked to perform as many alternating touches as possible for 15 seconds. Participants have three trials and one familiarization trial with 45 seconds rest between each trial. The average repetitions of the three attempts are recorded.

Shoulder function is assessed through the shoulder performance test (SPT). Participants are asked to start in a standing position with arm by side and to perform the following task: overhead reach, hand behind the head, and hand behind the back. We record the time in seconds taken to complete 20 repetitions of each task. Participants have one attempt with both upper limbs.

Furthermore, upper limb musculoskeletal disorders will be assessed using the Disabilities of the Arm, Shoulder, and Hand (DASH), which is comprised of a 30-item questionnaire designed to measure physical function and musculoskeletal disorders symptoms (55).

##### 2.5.3.7 Cognition

The Montreal Cognitive Assessment is used to assess global cognition and the NIH toolbox (www.nihtoolbox.org) is used to measure executive abilities, including response inhibition, cognitive flexibility, working memory, planning, insight, social cognition and behavior, and verbal fluency. This toolbox includes the Auditory Verbal Learning Test for immediate recall, Picture Sequence Memory Test for episodic memory, Oral Reading Recognition for Language, the Flanker for executive function and attention, the List Sorting test for working memory, Oral Symbol Digit Test for processing speed, Dimensional Change Card Sort Test for executive function, and Pattern Comparison for processing speed. We include an episodic memory composite that is derived from tests supported by the hippocampus and surrounding medial temporal lobe (56). This battery also includes memory tasks, Logical Reasoning, and Visual Reproductions.

##### 2.5.3.8 Patient-Reported Outcomes

We assess various aspects of psychosocial outcomes using questionnaires that have been validated and used in breast cancer patients, including the Functional Assessment of Cancer Therapy-Breast (FACT-B) for breast cancer-specific health-related quality of life (57), the Brief Fatigue Inventory (BFI) for the severity and impact of cancer-related fatigue (58), the Brief Pain Inventory (BPI) for a sensory and reactive dimension (59, 60), the Center for Epidemiologic Studies Depression scale (CES-D) for depression, the Pittsburg Sleep Quality Index (PSQI) for sleep quality (61, 62), and the State-Trait Anxiety Inventory (STAI) for anxiety (63). Recruitment and exercise adherence barriers and participants’ burden to the intervention are assessed using the Barriers to Recruitment Participation Questionnaire (BRPQ) (64), the Exercise Benefits/Barriers Scale (EBBS) (64, 65),, and the Perceived Research Burden Assessment (PRBA) (66), respectively.

##### 2.5.3.9 Physical Activity and Sedentary Behavior

Physical activity and sedentary behavior are assessed using the ActiGraph wGT3X-BT (*ActiGraph LLC, Pensacola, FL*) at all assessment timepoints. Participants are asked to wear the accelerometer on their hip for 7 consecutive days, except when showering or swimming. Wake wear time is used with a minimal data collection period of 4 days of at least 600 minutes per day for inclusion. We exclude non-wear time from the analyses, considering those accelerometer data where ≥90 minutes of consecutive zeros with a 2-minute spike tolerance (67). We use cutoff points that are commonly used among cancer survivors: sedentary time (<100 counts per minute), light physical activity (100–1951 counts per minute), and moderate-to-vigorous physical activity (≥1952 counts per minute) (68–70). ActiGraph data is analyzed using ActiLife software (*ActiLife 6; ActiGraph LLC*).

#### 2.5.4 Exploratory Outcomes

##### 2.5.4.1 Breast Cancer Outcomes

Breast cancer events are commonly used in lifestyle trials (71–75). Disease-free survival (DFS) is defined as the time from randomization to the documentation of the first of any of the following events: invasive ipsilateral breast tumor recurrence; local, regional, distant recurrence; invasive contralateral breast cancer; or ipsilateral and contralateral ductal carcinoma in situ, second primary invasive cancer (excluding carcinoma *in situ* and skin cancer other than melanoma). Overall survival (OS), distant DFS (DDFS), and recurrence-free interval (RFI) are included as exploratory endpoints. OS is defined as the time from randomization to death from any cause. DDFS is defined as events that are either lethal (death from any cause) or a direct threat to patient survival (distant recurrence or second primary invasive cancer). RFI is defined as events directly attributable to the original breast cancer including invasive ipsilateral breast tumor recurrence; local, regional, or distant recurrence; and death from breast cancer. These efficacy end points are assessed by reviewing medical records annually for 8 years from the time the participant enrolls in the study. Participants are called by phone to confirm their disease status or to assess their disease status if their medical records are unavailable.

#### 2.5.5 Exercise Adherence

Exercise adherence is captured for the TARE and CARE groups by the following measures: 1) percentage and the number of prescribed sessions attended (out of 48 sessions); 2) average minutes of exercise/week (76). Participants are considered compliant if they attend ≥ 80% of the total prescribed number of exercise sessions and ≥ 80% of the total prescribed minutes/exercise sessions. Reasons for any missed sessions are documented throughout the study, and a make-up session can be scheduled as soon as possible with no more than three supervised sessions allowed per week. Participants have a two-week window post the 16-week intervention to make up for any missed sessions due to potential illness, family or work obligations, and COVID-19-related issues. Any sessions not completed within the 18-week period is not made up and counted as missed session.

#### 2.5.6 Other Measures and Covariates

Patients’ sociodemographic, medical, health behavioral characteristics are collected using a self-reported questionnaire consisting of questions including demographics, socioeconomic status, medical conditions, concurrent medications, cancer treatment history, smoking status, and alcohol consumption. Dietary intake is assessed using the Automated Self-administered 24-hour (ASA-24) dietary assessment tool (77). Dietary intake is recorded by the participants at home on three days (i.e., two weekdays and one weekend day). Physical activity participation outside of the given intervention is assessed using an accelerometer (*Actigraph, Pensacola, FL*) at each assessment timepoint and a booklet of physical activity logs to record any form of daily structured exercise including type, duration, and intensity on a weekly basis, which is returned at the time of data collection visits or can be mailed in every four weeks.

### 2.6 Follow-Up Assessments

After the 16-week intervention, participants in the TARE and CARE groups will return 4 and 8 months after the post-intervention assessments to repeat the outcome measure testing. Medical record abstraction will occur for 8 years thereafter to assess prognostic outcomes (i.e. OS, DFS, etc.). During the follow-up period, participants will be encouraged to exercise independently, without study team supervision/intervention.

### 2.7 Adverse Events

Each patient is evaluated for potential adverse events (e.g., lymphedema, pain, muscle soreness, nausea, etc.) throughout the study period, which includes each biopsy procedure and each exercise session. Participants are provided with a phone number to contact study staff to report potential intervention-related adverse events. Participants are assessed and graded according to the NCI Common Terminology Criteria for Adverse Events (CTCAE) V5 and documented by the study personnel at each exercise session. Staff will report on study progress and injuries during weekly meetings either in person, by phone, or through weekly email updates. All adverse events, both serious and non-serious, and deaths that are encountered from initiation of study intervention, throughout the study, and within 30 days of the last study intervention sessions, should be followed to their resolution, or until the principal investigator assesses them as stable or determines the event to be irreversible, or the participant is lost to follow-up.

### 2.8 Sample Size

We anticipate recruiting 276 patients over a 48-month period and randomizing 92 patients to each arm of the study. Our hypotheses and analyses regarding our primary and secondary outcomes will reflect the comparison between CARE vs. TARE, and CARE vs. AC. Since the primary analysis involves two statistical comparisons, we will apply Bonferroni adjustment to control the overall type I error rate at 0.05 level. We estimated samples sizes for these two comparisons with each evaluated at two-sided alpha = 0.025. Our estimates of exercise intervention effect sizes were derived from randomized clinical trials (78–80) in addition to our preliminary data (81); most significantly, a study comparison of traditional endurance training vs. high-intensity circuit training (80) with effect sizes for outcomes common to our proposed trial (fat mass, lean body mass) was approximately 0.5-0.6. Effect sizes comparing exercise interventions to AC groups were larger, on 0.7-1.0. Our preliminary data compared exercise to AC (15 subjects per group), effect sizes were on the order of 1.0 (body composition measures) to 2.5-3.0 (inflammatory and other biomarkers) (81). The sample size determination was based on the comparison of the primary outcome between the CARE and TARE group because we expected that the difference between the CARE and AC group was larger than that between the CARE and TARE groups. We used an effect size of 0.5, two-sided alpha=0.025, and 80% power to estimate the sample size for comparisons of CARE vs. TARE, yielding a required 78 participants per group. To account for an anticipated 15% dropout rate, we will randomly assign 92 participants to each of the three groups. This will provide 80% or higher power to detect effect sizes of 0.5 and higher for other outcomes. For the optional biopsies, the sample size has been estimated at 50 participants per group based on our preliminary work (81). This will provide 80% power to detect a between-group difference, at a two-sided 0.025 alpha level, when the effect size is 0.9 for biopsy outcomes.

### 2.9 Statistical Analysis

Initial descriptive analyses will be used to evaluate baseline comparability among the three intervention groups, using ANOVA, or Kruskal-Wallis tests for continuous variables and chi-square tests for categorical variables. Unless otherwise specified, Statistical testing will be conducted at a two-sided 0.05 alpha level to determine statistical significance. For our primary and secondary outcomes, the variables used for the primary analysis will be the 2- and 4-month measurements of trial outcomes assessed during and at the end of the intervention period. Linear mixed-effects models will be used with the longitudinal data for the between-group comparisons, where two indicator variables for the treatment group (TARE, AC) relative to CARE, the baseline value of the outcome, and an indicator variable for measurement time (2-month/4-month) will be included as fixed-effects and subjects as random-effects in the models. With Bonferroni correction applied, the Wald test will be performed at 0.025 two-sided alpha level for each of the two comparisons regarding treatment effects (i.e., CARE vs. TARE, and CARE vs. AC); the between-group differences will be reported with corresponding 0.975 two-sided confidence intervals. Significant group differences will be followed by stratified analyses within measurement time (two and four months), and testing of group-time interactions, to evaluate treatment group differences within measurement time.

Post-intervention outcomes will be evaluated using the same linear mixed-effects models. The dependent variables will be the 8- and 12-month post-intervention measures, with the 4-month post-intervention measure, included as a fixed-effect.

The primary analysis would include all completed outcome assessments (regardless of whether the woman stayed on the intervention), inviting those who drop from the intervention to return for outcome assessments. The only missing data here would be those who drop the intervention and do not return for outcome assessments. Preliminary analyses will compare women who do and do not contribute to this analysis on baseline characteristics. Sensitivity/subgroup analyses will include (1): the receipt of chemotherapy as an established a-priori confounding factor due to possible chemotherapy-induced changes of chronic inflammation in adipose tissue (82–85), (2) multiple imputations of missing 2- and 4-month data (to evaluate the effect of missing data) (3); additional adjustment for baseline variables that differ between groups (to evaluate the effect of possible group differences on variables related to outcomes); and (4) adherence-based analysis, limiting subjects to those with at least 80% adherence (to estimate treatment effects under full adherence).

## 3 Discussion

The primary purpose of the AIM trial is to investigate if the CARE intervention is more effective than TARE or AC on obesity-associated chronic inflammation in post-treatment breast cancer survivors with central obesity. The AIM trial employs a comprehensive assessment of novel biomarkers of chronic inflammation using adipose tissue biopsies (e.g., M1, M2, and ATMs) and blood samples for systemic inflammation (e.g., TNF-α, IL-6, IL-8, and hs-CRP). Furthermore, we determine the effects of CARE on sarcopenic obesity, physical fitness and function, cognition, and quality of life, as the secondary and tertiary outcomes and breast cancer or clinical events as the exploratory outcomes. Lastly, we examine whether changes in inflammation can be maintained during an 8-month follow-up period without exercise programming and supervision.

Findings from the AIM trial will be highly relevant given that these results will contribute to further understanding exercise modalities and how they may improve adipose tissue inflammation implicated in poor prognosis in breast cancer survivors. The effects of exercise on inflammatory biomarkers associated with obesity-related breast cancer prognosis have been previously investigated in blood, however, little is known at the adipose tissue level in breast cancer survivors (86, 87). Excessive fat mass accumulation, especially at the visceral level, leads to adipose tissue dysfunction and a higher proportion of ATMs of the M1 phenotype (88). Adipose tissue dysregulation promotes an inflammatory state in which adipocytes and immune cells release pro-inflammatory cytokines and sex hormones, such as hs-CRP, IL-6, leptin, TNF-α, estrogens, among others (89, 90), increasing cell proliferation and cancer development (14). Indeed, breast cancer survivors have shown higher levels of circulating pro-inflammatory markers compared to the non-cancer population (91). The up-regulation of these markers is associated with the activation of the NF-kB pathway, which influences tumor growth and metastasis *via* increases in TNF-α and activation of STAT3 (activator of transcription) pathways increasing cell proliferation *via* IL-6 production (92), all contributors to poor prognosis (93, 94).

Individual side effects of obesity are commonly managed through pharmacological interventions for elevated blood pressure, triglycerides, and insulin resistance (95, 96). Targeting lifestyle interventions is critical in breast cancer survivors with obesity in order to prevent or manage chronic inflammation, particularly due to the physiologic burden of cancer-related pharmaceutical treatments (97). In this regard, exercise is a low-cost, non- pharmaceutical strategy to promote health outcomes and body composition in breast cancer survivors (98, 99). Exercise may reduce systemic inflammation in breast cancer survivors directly through the production of anti-inflammatory markers, or indirectly by decreasing fat mass (100), however these mechanisms are not completely understood yet (91, 101). We previously reported the effects of AE and RE on pro-inflammatory biomarkers among breast cancer survivors (102). In this study we found significant reductions in the exercise group compared with usual care in pro-inflammatory biomarkers including TNF-α, IL-6, IL-8, hs-CRP or leptin, and those related to insulin resistance (102). In contrast, some trials reported no effects of exercise on inflammatory biomarkers of breast cancer prognosis (103). A recent meta-analyses found significant reductions in CRP following AE and RE training, with no effects on other pro-inflammatory markers (86).

Furthermore, little is known regarding the effects of exercise on adipose tissue-specific inflammation in cancer survivors. To our knowledge, the AIM trial will be the first Phase II trial assessing the mechanisms by which different modalities of exercise (CARE vs. TARE) may impact adipose tissue-specific inflammation in breast cancer survivors with central obesity. This study was designed in part, by the results obtained from our previous randomized pilot trial (81), where we reported that 16-weeks of combined AE and RE training intervention can alter systemic and adipose tissue inflammation in breast cancer survivors (81). Specifically, we found reductions in the ratio of ATM M1, along with increases in the ratio of ATM M2 and anti-inflammatory cytokines such as adiponectin (81). Our findings align with previous studies that have examined changes in subcutaneous adipose tissue gene expression using lifestyle interventions in cancer primary prevention (104). Campbell et al. (105) showed that diet or exercise-induced weight loss resulted in changes in subcutaneous adipose-tissue expression in sex-hormone steroid synthesis, leptin, and insulin signaling as important pathways linking obesity, exercise, and breast cancer prevention. Additionally, Bruun et al. (106) found that a 15-week exercise intervention reduced adipose tissue inflammation, as determined by decreased gene expression of macrophage activation specific markers (CD14, CD68), IL-6, IL-8, and TNF-α in subcutaneous abdominal adipose tissue of men and women with obesity. Therefore, the lack of evidence from clinical based-trials examining the effects of exercise on adipose tissue-specific inflammation in breast cancer survivors establishes a need for study designs like the AIM trial. Given that current literature in this field is based on blood markers, exploring these biomarkers at the tissue level would allow us to better understand the role of exercise in metabolic dysregulation in breast cancer survivors.

A novel yet central aspect of the AIM trial is the exercise intervention design. Compared to TARE, CARE training is a novel exercise regimen as it combines the strengths of three different training components: 1) periodization, 2) circuit training, and 3) aerobic interval training, which has been individually effective in improving cardiometabolic and proinflammatory variables (20, 21, 107–109). Therefore, the CARE program challenges the current exercise prescription paradigm by combining linear periodization with an AE and RE training program performed in a circuit that progresses from low intensity to high-intensity interval training. Periodization is defined as a systematic progression of multiple training variables (e.g., intensity, volume, rest) with the aim of optimizing training-related outcomes (110). It is most commonly used in athletic program design but has been acknowledged as a potentially feasible and effective form of exercise prescription within various populations including cancer survivors (111, 112). Current literature is proposing the use of periodization in cancer patients to optimize physiological and physical outcomes (111, 113). In postmenopausal women, a 12-month periodized resistance training program increased lean mass and muscle strength and decreased body mass, fat mass, body fat %, and serum leptin levels (107). We have previously used a periodized resistance training program in prostate cancer survivors, and observed improvements in sarcopenia, body fat%, strength, and quality of life (114). In addition, emerging data show that a periodized exercise approach aligned with chemotherapy cycles was useful and well-tolerated by breast cancer patients during chemotherapy (115). However, its impact on adiposity, inflammation, and obesity in breast cancer survivors is unclear.

Furthermore, AE and RE are performed in a circuit to maximize time efficiency. Circuit training has been previously used in breast cancer survivors improving muscle, bone, and cardiorespiratory fitness (20, 21). Finally, aerobic interval training with short bouts at high intensity followed by brief recovery bouts at low intensity will also be integrated (116). Greater reductions in fat mass have been reported with high-intensity circuit training compared to low-intensity training (80), possibly given elevated metabolic rate in the hours following exercise cessation, referred to as excess post-exercise oxygen consumption (EPOC) (117). Increases in metabolism due to EPOC have been implicated in weight control and fat loss (118–121), in addition to improvements in cardiometabolic and proinflammatory factors. Compared to moderate-intensity continuous AE, high-intensity aerobic interval exercise has shown improvements in inflammatory biomarkers, body composition, and physical function in breast cancer survivors (109). Thus, our CARE program uses moderate-to-high intensity circuit training to optimize EPOC facilitated improvements in body composition and proinflammatory mediators. Therefore, this combination of periodization, circuit training, and aerobic interval training may be more effective to reduce systemic and adipose tissue-specific inflammation than traditional AE and RE in breast cancer survivors with central obesity.

Finally, the use of aerobic interval training will involve short bouts of exercise at a vigorous intensity, followed by brief, low-intensity recovery breaks (116). High-intensity aerobic interval training is well tolerated among breast cancer survivors (108) and has been demonstrated to elicit superior improvements in inflammatory biomarkers, body composition, and physical function when compared to moderate-intensity continuous AE (109). Thus, we aim to improve on aerobic interval exercise prescriptions for breast cancer survivors through its inclusion in CARE as a component within the periodized circuit training.

A major strength of the AIM trial is to target the understudied effects of different exercise modalities on adipose-tissue-specific inflammation. Additionally, supported by our use of a randomized controlled trial design that focuses on high-risk breast cancer survivors with central obesity and sedentary lifestyles. Furthermore, our novel exercise intervention is rigorously developed to optimize improvements in inflammation by incorporating periodization, circuit training, and aerobic interval training, and will be delivered in person by trained clinical exercise physiologists. With regard to limitations of the AIM trial, visceral adipose tissue may be more biologically active than subcutaneous adipose tissue, rendering the possibility that our analysis of subcutaneous adipose tissue inflammation will not capture all the potential benefits of exercise. Moreover, our supervised intervention may not be highly scalable due to the costs involving home exercise equipment and trainers supervising exercise sessions. Also, it’s possible for CARE and TARE to have different training doses because the intensity is pre-specified in CARE *via* the periodization, while it varies per individual in TARE due to the autoregulatory method of increasing intensity. Lastly, our population is only representative of the New England area and does not represent nationally or globally.

In conclusion, the AIM trial addresses an important research gap in the literature by assessing the effects of different exercise training modalities (CARE vs. TARE) on adipose tissue inflammation implicated in breast cancer prognosis. In this regard, although TARE alleviates systemic inflammation, metabolic diseases, and improves muscular strength in breast cancer survivors (102), we hypothesize that CARE will be more effective at reducing chronic inflammation through the inclusion of periodization, circuit training, and aerobic interval training. Therefore, results from this trial may depict the interrelated biomarkers involved in the associations between obesity, exercise, and breast cancer prognosis. If CARE is found to be an effective method for targeting chronic inflammation, this study may aid in the development of future exercise guidelines during cancer survivorship.

## Ethics Statement

The study protocol and patient consent form are approved by the Institutional Review Board at Dana-Farber Cancer Institute (IRB #20-172).

## Author Contributions

CD-C and SM conceptualized the study idea. CD-C, SM, and JD developed the study design and protocol. CD-C secured the funding of the trial. All authors contributed to manuscript writing, revision, and final approval.

## Funding

This study is supported by the National Cancer Institute (5R01CA214385).

## Conflict of Interest

The authors declare that the research was conducted in the absence of any commercial or financial relationships that could be construed as a potential conflict of interest.

## Publisher’s Note

All claims expressed in this article are solely those of the authors and do not necessarily represent those of their affiliated organizations, or those of the publisher, the editors and the reviewers. Any product that may be evaluated in this article, or claim that may be made by its manufacturer, is not guaranteed or endorsed by the publisher.
